# Soluble NKG2D ligand promotes MDSC expansion and skews macrophage to the alternatively activated phenotype

**DOI:** 10.1186/s13045-015-0110-z

**Published:** 2015-02-20

**Authors:** Gang Xiao, Xuanjun Wang, Jun Sheng, Shengjun Lu, Xuezhong Yu, Jennifer D Wu

**Affiliations:** 1 Department of Microbiology and Immunology, Medical University of South Carolina, Charleston, SC USA; 2 Key Lab for Puer Tea Science, Ministry of Education, Yunnan Agricultural University, Kunming, China; 3 Cancer Immunology Program, Hollings Cancer Center, Charleston, SC USA; 4 Present address: The Third Hospital of South Medical University, Guangzhou, China; 5 Present Address: Tongji Medical College, Huazhong University of Science and Technology, Wuhan, China

**Keywords:** NKG2D, Soluble MIC, MDSC, Macrophage, Tumor

## Abstract

Expression of surface NKG2D ligand MIC on tumor cells is deemed to stimulate NK and co-stimulate CD8 T cell anti-tumor immunity. Human cancer cells however frequently adopt a proteinase-mediated shedding strategy to generate soluble MIC (sMIC) to circumvent host immunity. High levels of sMIC have been shown to correlate with advanced disease stages in cancer patients. The underlying mechanism is currently understood as systemic downregulation of NKG2D expression on CD8 T and NK cells and perturbing NK cell periphery maintenance. Herein we report a novel mechanism by which sMIC poses immune suppressive effect on host immunity and tumor microenvironment. We demonstrate that sMIC facilitates expansion of myeloid-derived suppressor cells (MDSCs) and skews macrophages to the more immune suppressive alternative phenotype through activation of STAT3. These findings further endorse that sMIC is an important therapeutic target for cancer immunotherapy.

**Electronic supplementary material:**

The online version of this article (doi:10.1186/s13045-015-0110-z) contains supplementary material, which is available to authorized users.

## Background

Expression of surface NKG2D ligands on tumor cells is well proven to provoke tumor rejection through activation of NK and CD8 T cells in experimental animal models [1-3]. Human cancers however broadly evade this mechanism by adopting a protease-dependent shedding mechanism to generate soluble NKG2D ligands [4-6]. Among the many identified human NKG2D ligands [7], the MHC I chain-related molecule (MIC) is the best described and characterized ligands in human cancer. Elevated shedding of cell surface MIC has been associated with advanced disease stages and metastasis in many types of epithelial cancers [4,7-10]. It is well accepted that tumor-derived soluble MIC (sMIC) is a negative immune regulator in cancer patients, although the underlying mechanisms are not fully understood.

Multiple mechanisms have been uncovered by which soluble sMIC insults the immune system. Tumor-derived sMIC has been shown broadly downregulating NKG2D expression on NK, CD8 T, NKT, and γδ T cells and thus impair their effector function [4,10-12]. Very recently, we further show that tumor-derived sMIC perturbs NK cell periphery homoeostatic maintenance through disrupting the ability of NK cell to self-renew [13]. In this study, we report a novel mechanism by which tumor-derived sMIC negatively regulates host immunity and the immune reactive tumor microenvironment. With complementary *in vivo* and *in vitro* assays, we demonstrate that sMIC facilitates expansion of myeloid-derived suppressor cells (MDSCs) and skews macrophages to the alternative immune suppressive phenotype through activation of STAT3.

## Results and discussion

### sMICB increases frequency of MDSC and arginase I^+^ cells in bi-transgenic TRAMP/MIC mice

MIC is not expressed in rodents, which limits the potential to study the global impact of tumor-derived sMIC on host anti-tumor immunity *in vivo*. To overcome this limitation and to take the advantage that human MICB can serve as a mouse NKG2D ligand and activate mouse NKG2D [2,13-15], we have recently generated a bi-transgenic TRAMP/MICB spontaneous prostate tumor model in which the native form of human MICB was specifically expressed in the autochthonous transgenic adenocarcinoma mouse prostate (TRAMP) under the prostate-specific promoter [13]. The TRAMP/MICB model highly resembles human cancer patients in the kinetics of oncogenesis and tumor immunity in that MIC expression is concurrent with oncogenic events and MIC shedding correlates with tumor progression [13].

**Figure 1:**
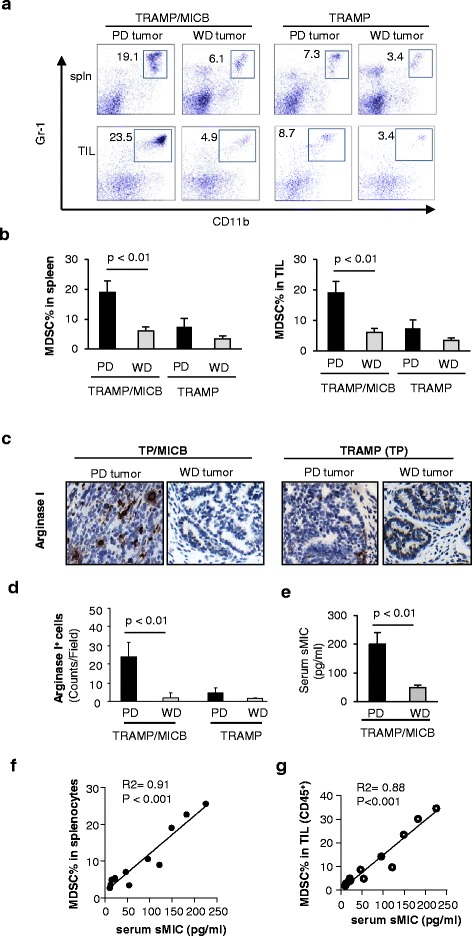
**Increased MDSC and arginase I+ cell population in the peripheral and tumor parenchyma in TRAMP/MIC mice is associated with elevated serum sMIC. (a)** Representative plot of flow cytometry analysis demonstrating the population of MDSC (CD11b^+^GR-1^+^) in the spleen and tumor parenchyma in TRAMP/MIC (MIC^+^) and TRAMP (MIC^−^) tumor-bearing TRAMP mice. **(b)** Summary data of MDSC percentage in splenocytes and tumor infiltrates. **(c)** Representative micrograph showing arginase I^+^ cells in the parenchyma of TRAMP/MIC and TRAMP tumors. **(d)** Summary data of arginase I^+^ cells in TRAMP/MIC and TRAMP tumors. **(e)** Serum levels of sMIC in TRAMP/MIC mice. **(f, g)** Correlation of serum levels of sMIC with numbers of MDSC in the splenocytes **(f)** and CD45^+^ tumor infiltrates **(g)** of TRAMP/MIC mice. A minimum of 10 TRAMP/MICB and TRAMP mice was analyzed. WD, well-differentiated. PD, poorly-differentiated.

In comparative studies of TRAMP/MICB mice with TRAMP littermates, we found that 1) TRAMP/MICB mice had significantly higher numbers of MDSC in the spleen and tumor infiltrates than their TRAMP littermates; 2) amongst the TRAMP/MICB animals, those with high levels of serum sMICB had significantly higher numbers of MDSC, a population generally defined as CD11b^+^GR-1^+^, in the spleen and tumor infiltrates (Figure 1a, b). We also found that significantly elevated number of cells that are arginase I^+^ in the TRAMP/MIC tumor infiltrates than in the TRAMP counterparts (Figure 1c, d). Moreover, among tumors from TRAMP/MICB mice, those with poorly differentiated (PD) tumors had significantly higher numbers of MDSC and arginase I^+^ cells in the tumor infiltrates than those with well-differentiated (WD) tumors (Figure 1a, d). Of note, the PD tumors are pathologically defined as invasive tumors with large mass [13,16,17]; whereas the WD tumors are pathologically organ-confined tumor with small mass [13,16,17] (Additional file 1: Figure S1). Notably, TRAMP/MIC mice with PD tumors also have significantly higher levels of serum sMIC than those with WD tumors (Figure 1e). Moreover, in TRAMP/MICB mice, the number of MDSCs in the spleen and tumor infiltrates significantly correlated with serum levels of sMIC (Figure 1f, g). Furthermore, neutralization of serum sMIC with a monoclonal antibody resulted in significant reduction of MDSCs in the spleen and TILs (Additional file 2: Figure S2). Given that arginase I is an emerging immune suppressive metabolic enzyme produced by MDSC and alternatively activated macrophages [18], these observations prompted us to investigate whether sMIC plays a role in the generation or expansion of MDSC and alternative macrophages.

### Intraperitoneal injection of sMIC promotes local accumulation of MDSCs and macrophages with alternative phenotype

MDSCs are a collective of heterogeneous population of immature myeloid cells (IMC) that are endowed with a robust immunosuppressive activity through secretion of immune suppressive factors, such as inducible nitric oxide synthase (iNOS), arginase, and indoleamine 2,3-dioxygnases (IDOs) [19]. The heterogeneous MDSC population are generally defined by surface marker CD11b^+^GR-1^+^ and considered to be immune suppressive in tumor host. MDSCs can be expanded during cancer development due to blockage of normal myeloid cell differentiation and maturation by tumor or tumor stroma-derived intrinsic factors [19].

**Figure 2:**
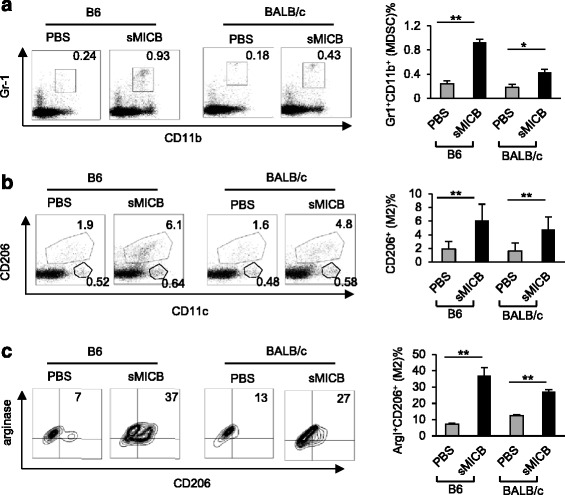
**Injection of sMICB promotes accumulation of MDSC and Arginase I+ macrophage in the peritoneal. (a)** Representative flow cytometry plots and summary data demonstrating the percentage of CD11b^+^Gr-1^+^ population in peritoneal exudate cells (PECs) harvested from B6 (*n* = 6) or BALB/c (*n* = 5) mice 3 days post i.p. injection with 200 μg sMICB. **(b)** Representative flow cytometry plots and summary data demonstrating expression of macrophage subtype marker CD206 (type II) and CD11c (type I) on gated F4/80^+^ PECs 6 days post sMICB injection. **(c)** Representative flow cytometry plots and summary data demonstrating arginase I expression in F4/80^+^CD206^+^ PECs. Data represent four independent experiments. *P < 0.05. **P < 0.01.

To provide evidence that sMIC contributes in part to the accumulation of MDSC in the TRAMP/MICB mice, we purified soluble MICB (sMICB) from 293 F-sMICB cell culture supernatant and intraperitoneally injected the sMICB into B6 or BALB/c mice (200 μg/mouse). As early as day three post-injection, a significantly higher numbers of cells with MDSC phenotype Gr-1^+^CD11b^+^ were found in the peritoneal fluid in mice that received sMICB injection than those having received control vehicle injection (Figure 2a). By day 6 post-sMIC injection, in addition to the significant increase in the number of cells expressing the MDSC phenotype (CD11b^+^Gr-1^+^), the number of cells that express markers for the alternative macrophage, F4/80^+^CD11c^low/−^CD206^+^ arginase I^+^, was also significantly increased in the PEC from mice that received sMICB injection in comparison to those having received control vehicle injection (Figure 2b, c; data not shown). A small percentage of F4/80^+^CD11c^+^CD206^−^ cells that would be considered as type I macrophage were also increased in association with sMICB injection, however not at a significant level (Figure 2b; data not shown). Notably, no significant amount of anti-MIC antibody was detected in the serum or peritoneal fluid at the given experimental time points nor were activation markers of lymphocyte subsets detected locally or systematically (data not shown). To further support that the *in vivo* effect is not due to unknown immune modulators resulted from sMICB purification process, we performed similar experiments with serum-free culture media, serum-free conditioned media from TRAMP-C2 cells bearing expression vector control, and serum-free conditioned media from TRAMP-C2 cells expressing sMICB. We obtained consistent results as with purified sMICB (Additional file 3: Figure S3). These data demonstrate that sMIC can facilitate the accumulation of CD11b^+^Gr-1^+^ cells and macrophages with alternative F4/80^+^CD206^+^ arginase I^+^ phenotype independent of tumor-derived intrinsic factors.

### sMICB promotes induction of MDSC *in vitro* through NKG2D and activation of STAT3

**Figure 3:**
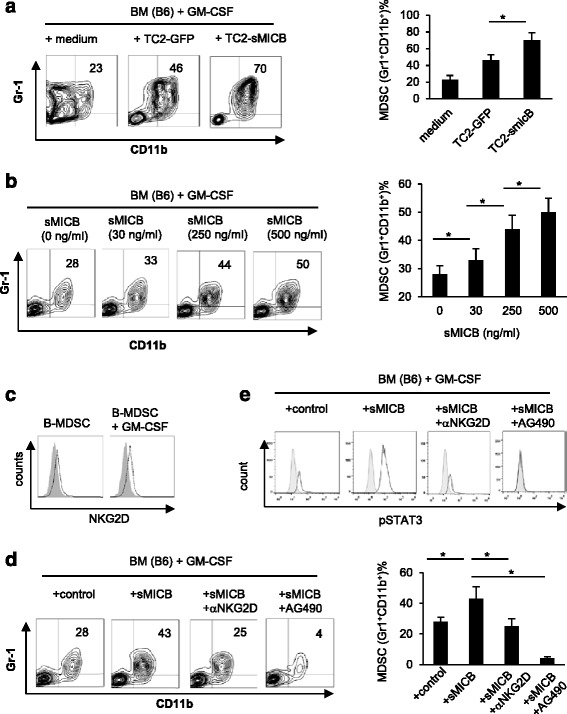
**sMIC promotes induction of MDSC through engagement of NKG2D and activation of STAT3. (a)** Bone marrow cells from B6 mice were co-cultured with sMICB expressing prostate tumor cell line TRAMP-C2 (TC2-sMICB-GFP, also as TC2-sMICB) or control TC2-GFP cells at a ratio of 10:1 for 3 days. Cells were harvested and stained with antibodies specific to CD45 (to differentiate myeloid cells from tumor cells), Gr-1, and CD11b for flow cytometry analyses. Data show representative plots and summary of CD11b^+^Gr-1^+^ in gated CD45^+^ cells. **(b)** Bone marrow cells from B6 mice were cultured in the presence of GM-CSF, with various concentration of purified sMICB for 3 days. Cells were harvested and analyzed for CD11b and Gr-1 expression. Data show representative plots and summary of the percentage of cells that are CD11b^+^Gr-1^+^. **(c)** Representative histogram showing NKG2D expression on myeloid cells with various culture conditions. Grey-filled profile, staining with isotype control. Open profile, staining with anti-NKG2D antibody CX5. **(d)** Bone marrow cells from B6 mice were cultured with GM-CSF in the presence or absence of sMICB (25 ng/ml) and with and without the NKG2D blocking antibody CX5 or STAT3 inhibitor AG490. Data show representative plots and summary of CD11b^+^Gr-1^+^ cells after 3 days of culture. **(e)** Levels of intracellular phosphorylated STAT3 in bone marrow myeloid cells cultured in the condition as described in **(d)**. Three replicates were performed in each experiment. Data represent five independent experiments. *P < 0.05.

We addressed whether the accumulation of MDSC induced by sMIC *in vivo* is a direct effect or an act through extrinsic cell mediators. We first co-cultured bone marrow cells isolated from wild type B6 or BALB/c mice with the mouse prostate tumor cell line TRAMP-C2 cells engineered to express sMICB (TC2-sMICB) or control TRAMP-C2 (TC2) cells that contain the expression vector only at various ratios in the presence of GM-CSF, a known growth factor for bone marrow myeloid progenitor cells and MDSC expansion [20]. After 3 days of co-culture, the number of CD11b^+^ Gr-1^+^ cells in the culture with PBS remained at a similar level to what was found in normal bone marrow contains [19] (20% to 30%, Figure 3a). When co-cultured with TC2 tumor cells, the number of CD11b^+^ Gr-1^+^ cells were significantly increased (46% ± 3.8%), consistent with current understanding that tumors can promote MDSC accumulation [19,21]. When co-cultured with TC2-sMICB cells, a further significant increase in the number of CD11b^+^ Gr-1^+^ cells was evident (70% ± 5.2%, Figure 3a). These observations suggest that sMICB may directly facilitate MDSC accumulation during myeloid cell differentiation.

We next addressed whether sMIC can induce MDSC accumulation *in vitro* in the absence of tumor cells. We cultured bone marrow cells with various concentrations of purified sMICB in the presence of GM-CSF and analyzed the cells at day 3 of culture. As representatively shown in Figure 3b, sMIC elicited a dose-dependent effect on the induction of Gr-1^+^CD11b^+^ cells. NKG2D, the only known cell surface receptor for sMIC, was detected on the surface MDSCs, with a trend of increased expression after exposure to GM-CSF (Figure 3c). We thus further asked whether NKG2D is necessary for the effect of sMIC in the current experimental setting. In the presence of the NKG2D blocking antibody CX5, sMIC failed to augment MDSC expansion (Figure 3d). This observation was substantiated by experiments demonstrating that sMIC has no effect on bone marrow cells from NKG2D^−/−^ mice (Additional file 4: Figure S4a). Together, these data confirmed a direct effect of sMIC on the accumulation of myeloid cells with MDSC phenotypes (Gr-1^+^CD11b^+^).

We sought to further understand the molecular pathways under which sMIC induces MDSC accumulation. MDSC expansion can be triggered by multiple factors that include cyclooxygenase-2(COX2), prostaglandins [22], stem cell factor (SCF) [23], macrophage colony-stimulating factor (M-CSF), IL-6 [24], GM-CSF [20], and vascular endothelial growth factors [25]. Signaling pathways triggered by most of these factors converge to the activation of the signal transducer and activator of transcription 3 (STAT3) [26], which is the main transcriptional factor regulating MDSC expansion [27,28]. MDSCs from tumor bearing mice showed markedly increased levels of phosphorylated STAT3 (pSTAT3) compared with IMCs from naïve mice [28]. As shown in Figure 3d, addition of the STAT3 inhibitor AG490 not only mitigated the effect of sMICB on MDSC accumulation but also nearly obliterated MDSC expansion (Figure 3d). Concurrently, the intracellular levels of pSTAT3 was decreased to base-level with anti-NKG2D blocking antibody CX5 and abolished with pSTAT3 inhibitor AG490 (Figure 3e). Given that GM-CSF induce potent STAT3 activation and that STAT3 is a key transcriptional factor in regulating MDSC expansion [19,20], the much more profound effect of AG490 than anti-NKG2D antibody on MDSC expansion and STAT3 is anticipated. No significant change in the levels of STAT3 phosphorylation was induced by sMICB in the same experiment settings with bone marrow cells from NKG2D^−/−^ mice [17] (Additional file 4: Figure S4b). Collectively, these data demonstrate that sMIC induces MDSC expansion through activation of STAT3 pathways.

### sMIC activation of STAT3 skews macrophage differentiation into to the alternative phenotype

**Figure 4:**
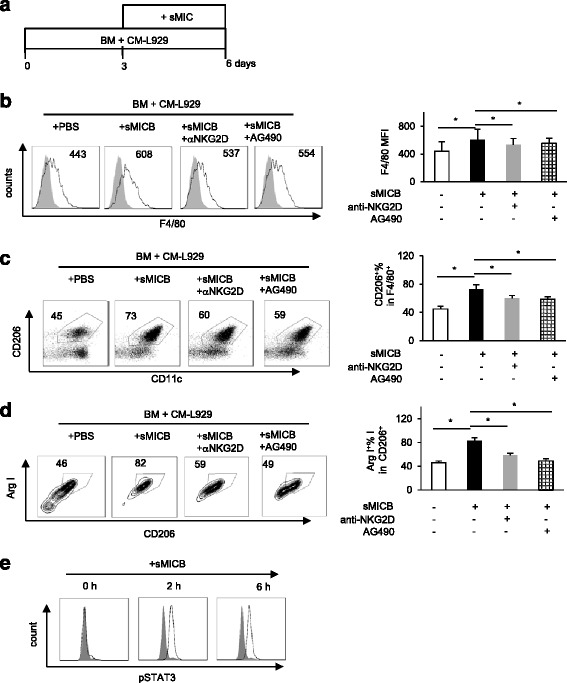
**sMICB skews macrophages into alternative activated phenotype through NKG2D and activation of STAT3.** B6 mice bone marrow cells were cultured in the presence of L929 conditioned media (CM-L929) for 3 days and cultured continually with or without sMICB in combination with NKG2D blocking antibody CX5 or STAT3 inhibitor AG490 for additional 3 days before harvest. Cells were stained with anti-CD206, anti-CD11c, and anti-F4/80 in combination with intracellular Arginase I staining. **(a)** Schema of bone marrow (BM) culture condition. **(b, c, d)** Representative histogram and summary data showing that sMIC increases the expression of F4/80 **(b)**, CD206 in gated F4/80^+^ cells **(c)**, and arginase I (arg I) in CD206^+^ cells **(d)**. Data also show that blocking NKG2D or STAT3 mitigates the effect of sMICB. **(e)** Intracellular levels of pSTAT3 in BM-derived macrophages at various time points post-exposure to sMICB. Three replicates were performed in each experiment. Data represent five independent experiments. *P < 0.05.

We have observed increased arginase I expression in the tumor tissue from our TRAMP/MIC mice that have high levels of circulating sMIC and also in mice that were injected with purified sMIC. We thus sought to ask whether prolonged sMIC exposure can skew myeloid cells into an immune suppressive arginase I^+^ alternative phenotype during differentiation. We cultured bone marrow cells isolated from B6 mice with conditioned media harvested from L929 cells (CM-L929) to allow macrophage generation for 3 days [29]. With the addition of 25 ng/ml of sMICB to the media at day 3 and allowing cells to continue differentiation for additional 3 days (Figure 4a), we found that addition of sMICB in the culture significantly increased the level of F4/80 expression (Figure 4b). When the F4/80^+^ population were further analyzed, the number of cells that express the scavenger receptor CD206, a marker for the alternative phenotype of macrophage, was significantly increased with inclusion of sMICB (Figure 4c). Moreover, these CD206^+^ cells also express arginase I (Figure 4d), confirming the phenotype of the alternatively activated macrophages. Blocking NKG2D with the antibody CX5 or inhibiting STAT3 activation with AG490 similarly diminished the impact of sMICB (Figure 4b, c, d), indicating that sMICB acts through NKG2D and STAT3 pathway to skew macrophage into the alternatively activated phenotype. This conclusion was confirmed by experiments demonstrating that sMICB had no effect on macrophage differentiation originated from NKG2D^−/−^ bone marrow cells (Additional file 4: Figure S4c). To confirm that the STAT3 pathway is activated by sMICB during macrophage differentiation, we assessed levels of intracellular pSTAT3 and showed that STAT3 was phosphorylated in macrophage as early as 2 h after exposure to sMICB and the level of pSTAT3 persists with the presence of sMICB (Figure 4e). We obtained consistent results from bone marrow cells isolated from BALB/c mice (Additional file 5: Figure S5). Given that the expression of NKG2D is detected in activated macrophages, these data suggest that activation of STAT3 signaling via sMIC/NKG2D interaction can promote macrophage polarization into a more immune suppressive alternative phenotype. Notably, activation of STAT6 can also promote macrophage differentiation into the alternatively activated phenotype [30]. We observed a base level of pSTAT6 in the macrophages with CM-L929 media; however, the level was not influenced by sMIC (data not shown). These observations provided an explanation of the sustained percentage of ArgI^+^CD206^+^ cells with the STAT3 inhibitor AG490 (Figure 4d).

To this end, we demonstrate that tumor-derived soluble NKG2D ligand sMICB facilitates the expansion of MDSC and skews macrophage differentiation into an alternatively active phenotype through NKG2D and activation of the STAT3 pathway. Classically, activation of a PI3K/Grb2-Vav pathway is the canonical outcome of NKG2D signaling via coupling to the adaptor molecule DAP10 in both mouse and human cells [31,32], although NKG2D in mouse, ambiguously in human, has been shown to promiscuously and selectively associate with DAP10 or DAP12 adaptor molecule, depending on the availability of the adaptor partners, cell types, and cell activation status [33-36]. Whether NKG2D signaling in MDSC or activated macrophage primarily activates the PI3K pathway with subsequent activation of STAT3 pathways or directly activates the STAT3 pathway is an interesting question to be further investigated. It has been shown that PI3K signaling can bisect at phosphatidylinositol 3,4,5-trisphosphate (PIP3) to activate the non-receptor bone-marrow tyrosine kinase BTK and the downstream STAT3 signaling during cellular oncogenic transformation [37]. Provided with this knowledge, it is reasonable to speculate that activation of STAT3 pathways via NKG2D signaling in the bone marrow-originated MDSCs and macrophages lies downstream of the canonical PI3K signaling. It would be an interesting future investigation to test this speculation with specific PI3K or BTK inhibitors.

## Conclusion

Tumor-derived soluble NKG2D ligands, namely sMIC, is known to negatively impact host immune response by downregulating NKG2D expression on effector NK and CD8 T cells and most recently shown by perturbing NK cell peripheral maintenance. Here we demonstrate that sMIC can also promote the expansion of MDSC and skew macrophage into a more immune suppressive phenotype in a given microenvironment. We demonstrated that sMIC can activate STAT3 pathway in cells of myeloid lineage to foster their immune suppressive potential. We have demonstrated a novel mechanism by which tumor-derived sMIC may temper the immune reactive tumor microenvironment. Our findings further emphasize the significance of targeting sMIC for cancer immune therapy.

## Methods

### Reagents, constructs, and cell lines

STAT3 inhibitor AG490 was purchased from Invivogen (San Diego, CA, USA). cDNA containing the α1-3 ectodomain of MICB fused with HIS6-Tag at the C-terminus was synthesized by GENEWIZ Inc. (South Plainfield, NJ, USA) and subcloned into the retroviral expression vector pBMN-GFP as described previously [14]. The sMICB expression plasmid pBMN-GFP-sMICB or control vector pBMN-GFP was transfected into 293 T cells. After 2 weeks of selection with puromycin (5 μg/ml), GFP-positive cells were isolated by flow cytometry sorting. Serum-free culture supernatant of sorted GFP^+^ 293 T cells was collected and subjected to Ni-sepharose Fast Flow column from (GE Healthcare, Pewaukee, WI, USA) and eluted with imidazole gradient increase method according to manufacturer’s instruction. The elution was loaded onto the Hydrophobic Interaction Chromatography (HIC) column (GE Healthcare) for size fractionation. The final collected fraction was buffer exchange with the PBS (pH 7.2). The purity of sMICB after buffer exchange was assayed by SDS-PAGE gel and Western-blotting with anti-MIC antibody (Additional file 6: Figure S6). Specific binding of purified recombinant sMICB to mouse NKG2D was confirmed by flow cytometry analyses (Additional file 6: Figure S6). Generation of sMICB-expressing TRAMP-C2 cells was described previously [14]. Tumor cell lines TRAMP-C2, derivatives, and mouse fibroblast L929 cell line were maintained in Dulbecco modified Eagle medium (DMEM) plus 10% fetal bovine serum and antibiotics. Conditioned media of L929 cells was collected, centrifuged, and filtered through a 0.22-μm filter before being applied to bone marrow culture.

### Mice and *in vitro* bone marrow differentiation

All animal procedures were approved by the MUSC Institutional Animal Care and Use Committee. 7- to 10-week-old male C57BL/6 and BALB/c mice were purchased from The Jackson Laboratory (Bar Harbor, ME, USA). NKG2D-deficient mice (gift of Dr. David Raulet, University of California at Berkeley) were bred in house. All mice were housed in Specific Pathogen Free facility. Single cell suspension of bone marrow (BM) cells were plated at the density of 3 × 10^6^ cells/ml in DMEM supplemented with 10% fetal bovine serum, 50 mM 2-mercaptoethanol, 10 mM 4-(2-hydroxyethyl)-1-piperazineethanesulfonic acid buffer (Life technologies, Grand Island, NY, USA), 1 mM sodium pyruvate, 100 U/ml penicillin, 100 mg/ml streptomycin, and amino acids (1.5 mM L-glutamine, L-arginine, and L-asparagine), with or without 1 ng/ml rmGM-CSF (Biolegend, San Diego, CA, USA). sMICB protein of designated concentration was added where indicated. In some experiments, bone marrow cells were cultured with conditioned media derived from L929 cell lines.

### Peritoneal cell isolation

Mice were injected via intraperitoneal (i.p) routine with sMICB or control PBS flow through from the culture supernatant of 293 F cells that were transduced with control vector expressing HIS6-Tag alone. At indicated time points, animals were euthanized and the peritoneal exudate cells (PECs) were harvested by lavage with 10 ml of ice-cold PBS.

### Flow cytometry

Cells were stained using combination of fluorochrome-conjugated anti-mouse CD11b and Gr-1 antibody to define the general population of MDSC and fluorochrome-conjugated anti-mouse F4/80 in combination with CD206 and CD11c and intracellular staining of arginase I to define the phenotype of macrophage. All fluorescence-conjugated antibodies were from eBioscience except for rabbit anti-pSTAT3 (Cell signaling, Danvers, MA, USA). Appropriately conjugated species and isotype-matched IgG was used as staining controls. Multi-colored flow cytometry data were collected on a BD LSRII flow cytometer and analyzed with FlowJo software (Tree Star).

### Statistical analysis

All results are expressed as the mean ± SEM. Differences between the mean of groups were analyzed using student’s *t*-test. *P* < 0.05 was considered as significant.

## Additional files

Additional file 1: Figure S1. Prostate weight in TRAMP and TRAMP/MICB animals. This data has been published partially (references 13, 16, 17). The poorly-differentiated (PD) and well-differentiated (WD) tumors were defined according to pathology criteria. Generally, PD tumors are invasive tumors.

Additional file 2: Figure S2. Representative flow cytometry plots demonstrating that neutralizing circulating sMIC with a monoclonal antibody reduces the population of MDSC in the spleen and tumor infiltrates (TILs). Data represents at least five animals in the control or anti-sMIC treated group.

Additional file 3: Figure S3. Conditioned culture medium (CM) from TRAMP-C2-sMICB (TC2-sMICB) cells induce expansion of MDSC *in vivo*. B6 mice were injected i.p. with 200 μl of control serum-free culture media, conditioned media collected from TRAMP-C2 mouse prostate tumor cells with expression vector control (TC2-Vector) or conditioned media collected from TC2-sMICB cells at day 1 and day 3. Mice were sacrificed at day 6. PEC were collected and analyzed for the MDSC. Data represent five animals in each experiment. The experiments were repeated three times and consistent results were obtained.

Additional file 4: Figure S4. sMICB has no effect on NKG2D-deficient myeloid MDSC expansion or macrophage polarization. Bone marrow (BM) cells from wild-type or NKG2D^−/−^ B6 mice were cultured in MDSC/macrophage differentiation media (L929-CM) supplemented with control flow-through from 293 T supernatant or purified sMICB (50 ng/ml). At day 3 of culture, cells were analyzed for MDSC population and STAT3 phosphorylation in MDSC (**a, b**). At day 6 of culture, cells were analyzed for macrophage (gated on F4/80^+^) phenotypes (**c, d**). Data represents three independent experiments with three replicates in each experiment.

Additional file 5: Figure S5. sMICB skews macrophages into alternative phenotype through NKG2D and activation of STAT3 in BALB/c background. Bone marrow cells from BALB/c mice were cultured in the presence of L929 conditioned media (CM-L929) for 3 days and cultured continually with or without sMICB in combination with NKG2D blocking antibody CX5 or STAT3 inhibitor AG490 for additional 3 days before harvest. Cells were stained with anti-CD206, anti-CD11c, and anti-F4/80 in combination with intracellular arginase I staining. **(a, b)** Representative histogram and summary data showing that sMIC increases the expression of CD206 in gated F4/80^+^ cells (a), and arginase I (Arg I) in gated CD206^+^ cells (b). Data also show that blocking NKG2D or STAT3 mitigates the effect of sMICB. Data represent five independent experiments. *P < 0.05.

Additional file 6: Figure S6. Validation of purified sMICB. **(a)** Coomassie blue staining of SDS gel of sMICB elution after PBS buffer exchange. **(b)** Western-blot probed with anti-MIC antibody H-300 (Santa Cruz) and HRP-conjugated donkey anti-rabbit secondary antibody. Bioactive sMIC produced in mammalian system is highly glycosylated. Lane 1, without PNGaseF de-glycosylation. Lane 2, after PNGaseF de-glycosylation. **(c)** Histogram of flow cytometry showing specific binding of purified sMICB to mouse NKG2D on mouse NK1.1 (mNK1.1) and MDSC (mMDSC). Mouse (B6) splenic NK cells or bone marrow-derived MDSCs were stained with purified sMICB (HIS-Tagged) (20 μg/ml) followed by rabbit anti-HIS antibody and PE-conjugated goat anti-rabbit antibody. Dark grey profile represents sMICB binding. Black profile represents reduced sMICB binding when cells were pre-incubated with NKG2D blocking antibody CX5 (10 μg/ml). Light grey profile represents control rabbit IgG staining.
