# Supplementary material for: Soluble NKG2D ligand promotes MDSC expansion and skews macrophage to the alternatively activated phenotype
Source: J Hematol Oncol. 2015 Feb 20;8:13. doi: 10.1186/s13045-015-0110-z (PMC4342005; doi:10.1186/s13045-015-0110-z)
Supplement: Supplementary file 1 — Prostate weight in TRAMP and TRAMP/MICB animals. This data has been published partially (references 13, 16, 17). The poorly-differentiated (PD) and well-differentiated (WD) tumors were defined according to pathology criteria. Generally, PD tumors are invasive tumors. [file 13045_2015_110_MOESM1_ESM.pdf]

Supplement Figure 1

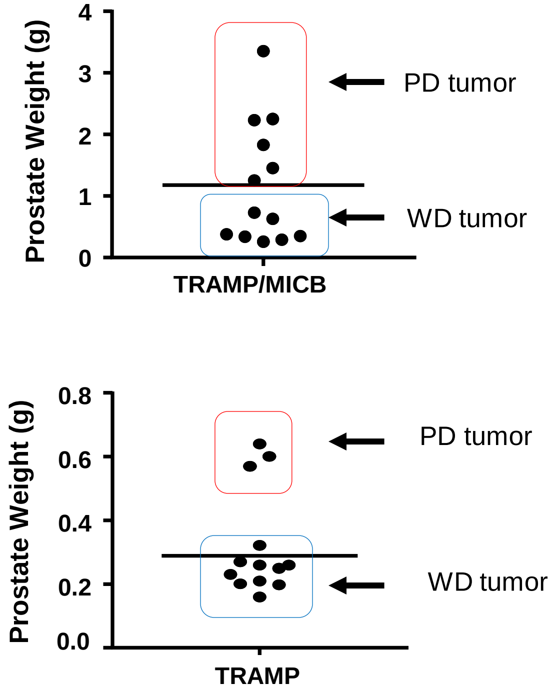

Figure S1. Prostate weight in TRAMP and TRAMP/MICB animals. Partial of this data has been published (references 13, 16, 17). The poorly-differentiated (PD) and well-differentiated (WD) tumor were defined according to pathology criteria. Generally, PD tumors are invasive tumors.
