# Supplementary material for: Soluble NKG2D ligand promotes MDSC expansion and skews macrophage to the alternatively activated phenotype
Source: J Hematol Oncol. 2015 Feb 20;8:13. doi: 10.1186/s13045-015-0110-z (PMC4342005; doi:10.1186/s13045-015-0110-z)
Supplement: Supplementary file 2 — Representative flow cytometry plots demonstrating that neutralizing circulating sMIC with a monoclonal antibody reduces the population of MDSC in the spleen and tumor infiltrates (TILs). Data represents at least five animals in the control or anti-sMIC treated group. [file 13045_2015_110_MOESM2_ESM.pdf]

Supplement Figure 2

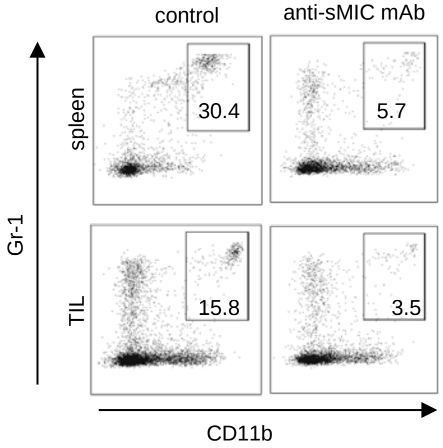

Figure S2. Representative flow cytometry plots demonstrating that neutralizing circulating sMIC with an monoclonal antibody reduces the population of MDSC in the spleen and tumor infiltrates (TILs). Data represents at least 5 animals in the control or anti-sMIC treated group.
