# Supplementary material for: Soluble NKG2D ligand promotes MDSC expansion and skews macrophage to the alternatively activated phenotype
Source: J Hematol Oncol. 2015 Feb 20;8:13. doi: 10.1186/s13045-015-0110-z (PMC4342005; doi:10.1186/s13045-015-0110-z)
Supplement: Supplementary file 3 — Conditioned culture medium (CM) from TRAMP-C2-sMICB (TC2-sMICB) cells induce expansion of MDSC in vivo. B6 mice were injected i.p. with 200 μl of control serum-free culture media, conditioned media collected from TRAMP-C2 mouse prostate tumor cells with expression vector control (TC2-Vector) or conditioned media collected from TC2-sMICB cells at day 1 and day 3. Mice were sacrificed at day 6. PEC were collected and analyzed for the MDSC. Data represent five animals in each experiment. The experiments were repeated three times and consistent results were obtained. [file 13045_2015_110_MOESM3_ESM.pdf]

Supplement Figure 3

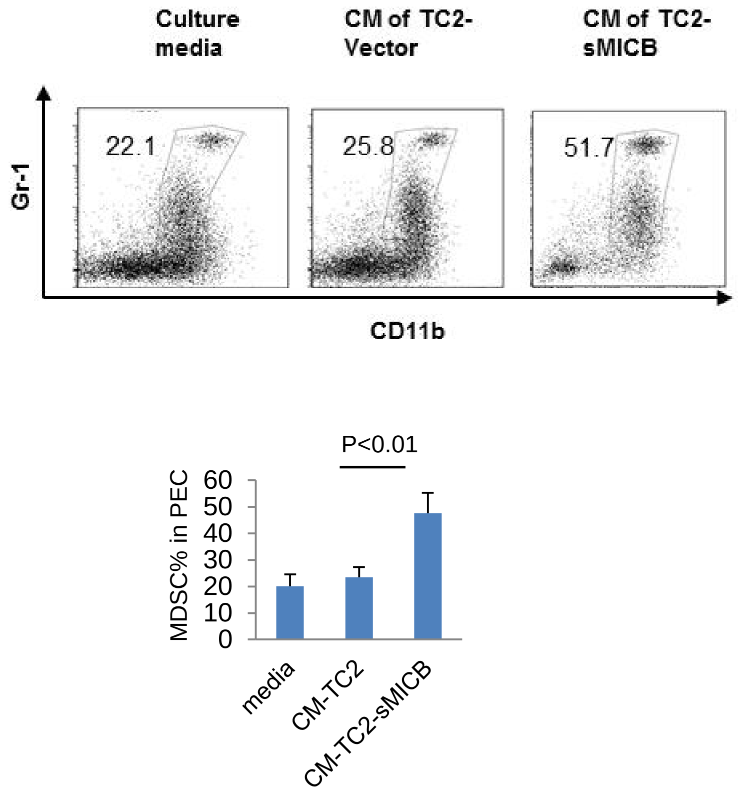

Figure S3. Conditioned culture medium (CM) from TRAMP-C2-sMICB (TC2-sMICB) cells induce expansion of MDSC *in vivo*. B6 mice were injected i.p. with 200 µl of control serum-free culture media, conditioned media collected from TRAMP-C2 mouse prostate tumor cells with expression vector control (TC2-Vector), or conditioned media collected from TC2-sMICB cells at Day 1 and Day 3. Mice were sacrificed at day 6. PEC were collected and analyzed for the MDSC. Data represent 5 animals in each experiment. The experiment were repeated 3 times and consistent results were obtained.
