# Supplementary material for: Soluble NKG2D ligand promotes MDSC expansion and skews macrophage to the alternatively activated phenotype
Source: J Hematol Oncol. 2015 Feb 20;8:13. doi: 10.1186/s13045-015-0110-z (PMC4342005; doi:10.1186/s13045-015-0110-z)
Supplement: Supplementary file 4 — sMICB has no effect on NKG2D-deficient myeloid MDSC expansion or macrophage polarization. Bone marrow (BM) cells from wild-type or NKG2D−/− B6 mice were cultured in MDSC/macrophage differentiation media (L929-CM) supplemented with control flow-through from 293 T supernatant or purified sMICB (50 ng/ml). At day 3 of culture, cells were analyzed for MDSC population and STAT3 phosphorylation in MDSC (a, b). At day 6 of culture, cells were analyzed for macrophage (gated on F4/80+) phenotypes (c, d). Data represents three independent experiments with three replicates in each experiment. [file 13045_2015_110_MOESM4_ESM.pdf]

Supplement Figure 4

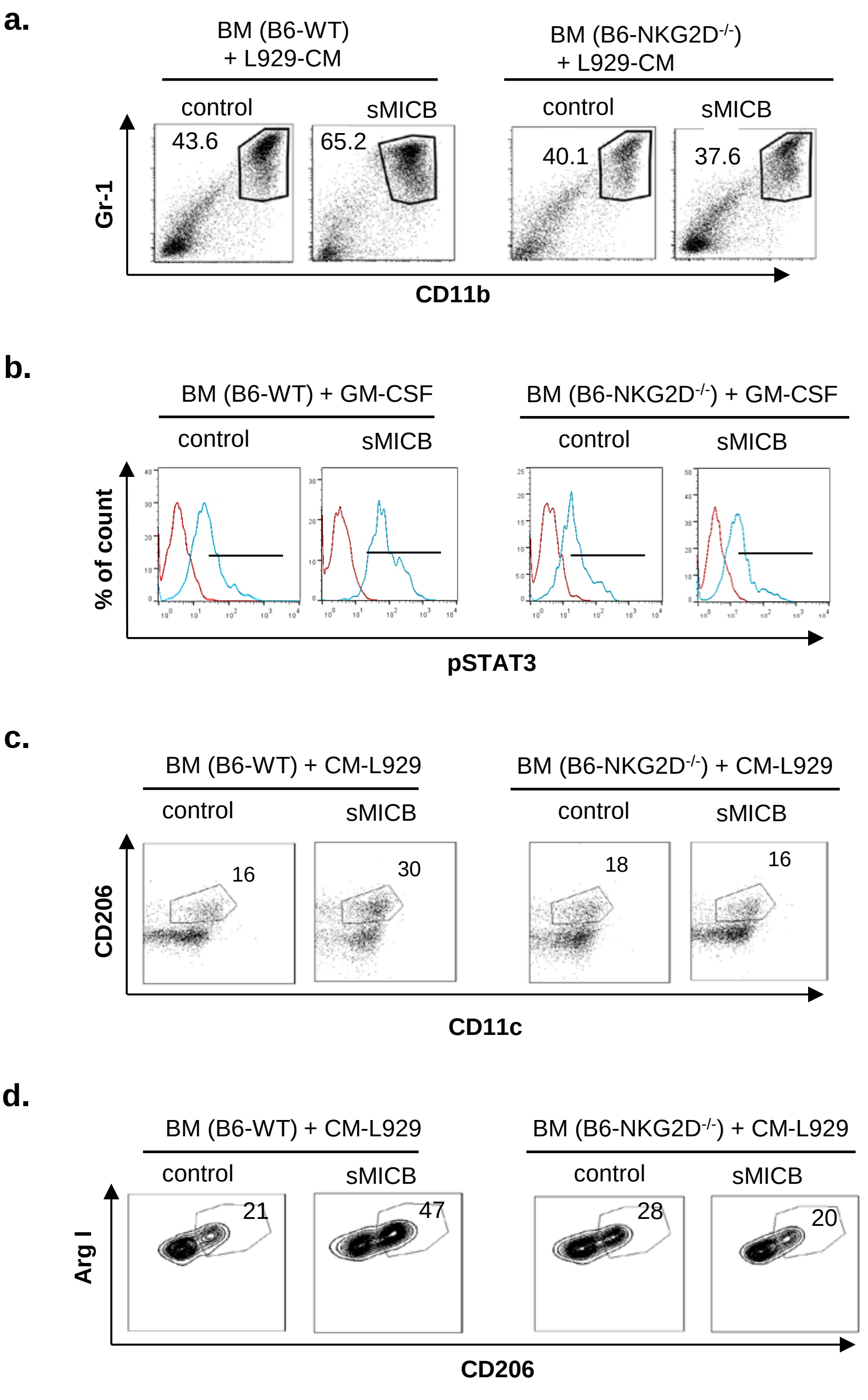

Figure S4. sMICB has no effect on NKG2D-deficient myeloid MDSC expansion or macrophage polarization. Bone marrow (BM) cells from wild-type or NKG2D$^{-/-}$ B6 mice were cultured in MDSC/macrophage differentiation media (L929-CM) supplemented with control flow-through from 293T supernatant or purified sMICB (50 ng/ml). At day 3 of culture, cells were analyzed for MDSC population and STAT3 phosphorylation in MDSC (**a** and **b**). At day 6 of culture, cells were analyzed for macrophage (gated on F4/80^{+}) phenotypes (**c** and **d**). Data represents three independent experiments with three replicates in each experiment.
