# Supplementary material for: Soluble NKG2D ligand promotes MDSC expansion and skews macrophage to the alternatively activated phenotype
Source: J Hematol Oncol. 2015 Feb 20;8:13. doi: 10.1186/s13045-015-0110-z (PMC4342005; doi:10.1186/s13045-015-0110-z)
Supplement: Supplementary file 5 — sMICB skews macrophages into alternative phenotype through NKG2D and activation of STAT3 in BALB/c background. Bone marrow cells from BALB/c mice were cultured in the presence of L929 conditioned media (CM-L929) for 3 days and cultured continually with or without sMICB in combination with NKG2D blocking antibody CX5 or STAT3 inhibitor AG490 for additional 3 days before harvest. Cells were stained with anti-CD206, anti-CD11c, and anti-F4/80 in combination with intracellular arginase I staining. (a, b) Representative histogram and summary data showing that sMIC increases the expression of CD206 in gated F4/80+ cells (a), and arginase I (Arg I) in gated CD206+ cells (b). Data also show that blocking NKG2D or STAT3 mitigates the effect of sMICB. Data represent five independent experiments. *P < 0.05. [file 13045_2015_110_MOESM5_ESM.pdf]

Supplement Figure 5

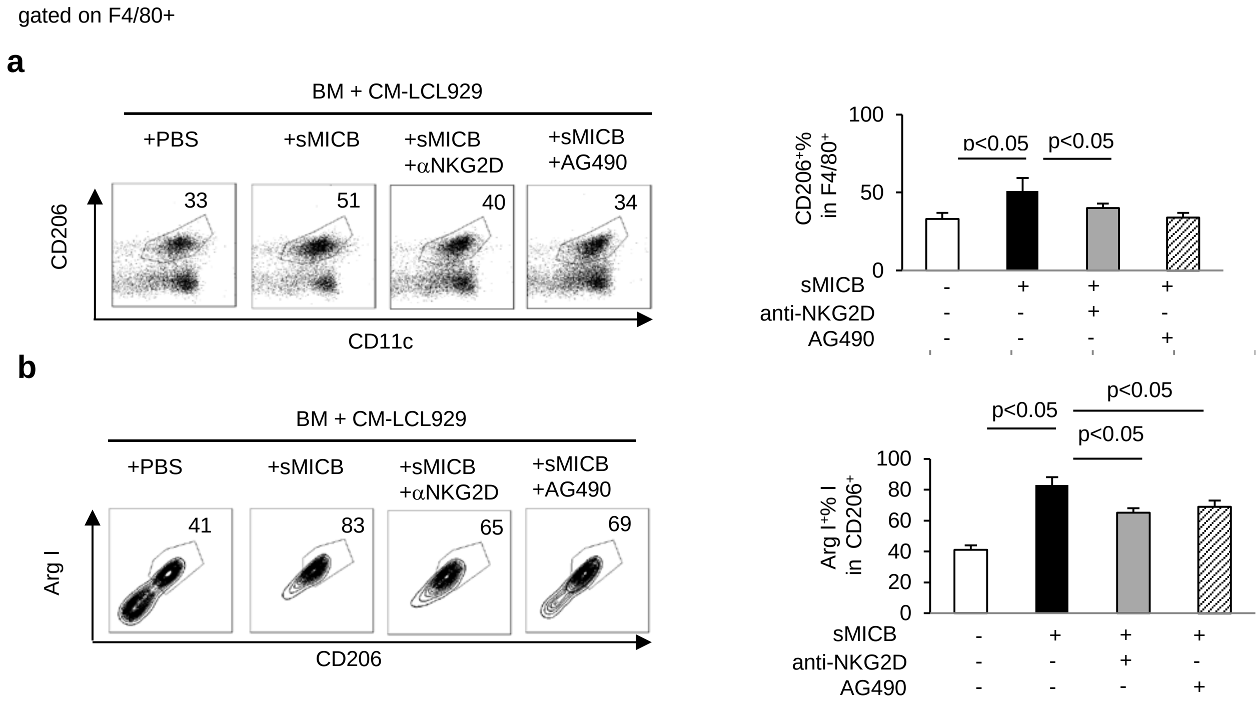

Figure S5. sMICB skews macrophages into alternative phenotype through NKG2D and activation of STAT3 in balb/c background. Bone marrow cells from Balb/c mice were cultured in the presence of L929 conditioned media (CM-L929) for 3 day and continue to culture with or without sMICB in combination with NKG2D blocking antibody CX5 or STAT3 inhibitor AG490 for additional 3 days before harvest. Cells were stained with anti-CD206, anti-CD11c and anti-F4/80 in combination with intracellular Arginase I staining. **a** and **b**. Representative histogram and summary data showing that sMIC increases the expression of CD206 in gated F4/80^{+} cells (**a**), and Arginase I (Arg I) in gated CD206+ cells (**b**). Data also show that blocking NKG2D or STAT3 mitigates the effect of sMICB. Data represent five independent experiments. *, $p<0.05$.
