# Supplementary material for: Soluble NKG2D ligand promotes MDSC expansion and skews macrophage to the alternatively activated phenotype
Source: J Hematol Oncol. 2015 Feb 20;8:13. doi: 10.1186/s13045-015-0110-z (PMC4342005; doi:10.1186/s13045-015-0110-z)
Supplement: Supplementary file 6 — Validation of purified sMICB. (a) Coomassie blue staining of SDS gel of sMICB elution after PBS buffer exchange. (b) Western-blot probed with anti-MIC antibody H-300 (Santa Cruz) and HRP-conjugated donkey anti-rabbit secondary antibody. Bioactive sMIC produced in mammalian system is highly glycosylated. Lane 1, without PNGaseF de-glycosylation. Lane 2, after PNGaseF de-glycosylation. (c) Histogram of flow cytometry showing specific binding of purified sMICB to mouse NKG2D on mouse NK1.1 (mNK1.1) and MDSC (mMDSC). Mouse (B6) splenic NK cells or bone marrow-derived MDSCs were stained with purified sMICB (HIS-Tagged) (20 μg/ml) followed by rabbit anti-HIS antibody and PE-conjugated goat anti-rabbit antibody. Dark grey profile represents sMICB binding. Black profile represents reduced sMICB binding when cells were pre-incubated with NKG2D blocking antibody CX5 (10 μg/ml). Light grey profile represents control rabbit IgG staining. [file 13045_2015_110_MOESM6_ESM.pdf]

Supplement Figure 6

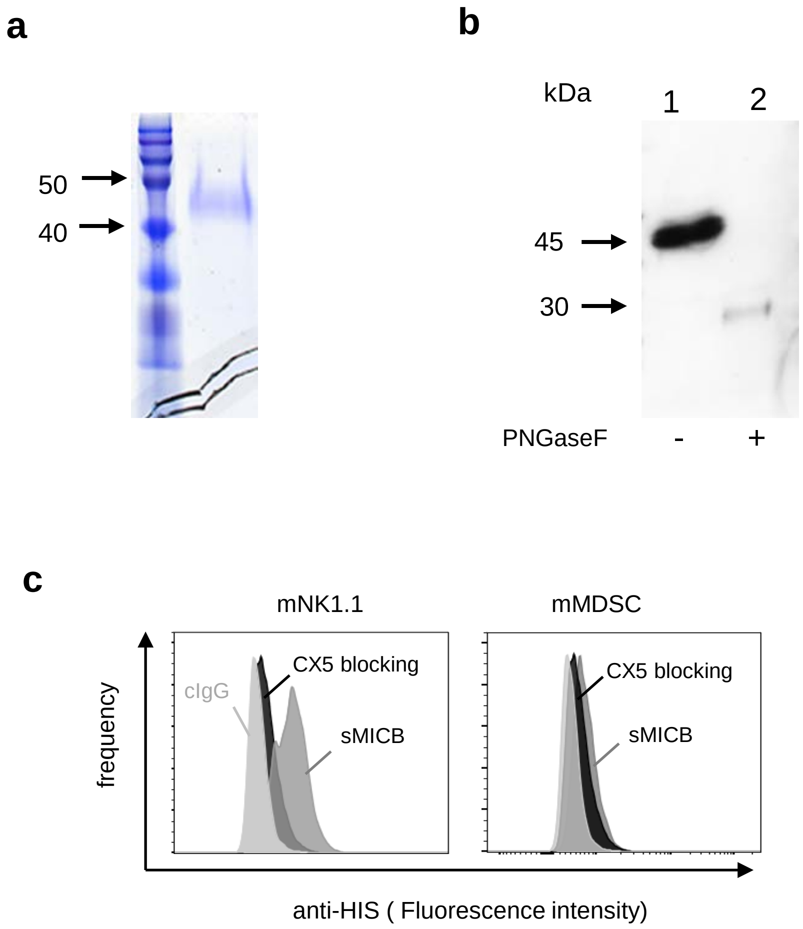

Figure S6. Validation of purified sMICB. a, Coommasie blue staining of SDS gel of sMICB elution after PBS buffer exchange. **b,** Western-blot probed with anti-MIC antibody H-300 (Santa Cruz) and HRP-conjugated donkey anti-rabbit secondary antibody. Bioactive sMIC produced in mammalian system is highly glycosylated. Lane 1, without PNGaseF de-glycosylation. Lane 2, after PNGaseF de-glycosylation. **c**, Histogram of flow cytometry showing specific binding of purified sMICB to mouse NKG2D on mouse NK1.1 (mNK1.1) and MDSC (mMDSC). Mouse (B6) splenic NK cells or bone marrow-derived MDSCs were stained with purified sMICB (HIS-Tagged) (20 µg/ml) followed by rabbit anti-HIS antibody and PE-conjugated goat anti-rabbit antibody. Dark grey profile represents sMICB binding. Black profile represents reduced sMICB binding when cells were pre-incubated with NKG2D blocking antibody CX5 (10 µg/ml). Light grey profile represents control rabbit IgG staining.
